# Discovery of Novel Conotoxin Candidates Using Machine Learning

**DOI:** 10.3390/toxins10120503

**Published:** 2018-12-01

**Authors:** Qing Li, Maren Watkins, Samuel D. Robinson, Helena Safavi-Hemami, Mark Yandell

**Affiliations:** 1 Eccles Institute of Human Genetics, University of Utah, Salt Lake City, UT 84112, USA; liqing850104@gmail.com; 2 Huntsman Cancer Institute, University of Utah, Salt Lake City, UT 84112, USA; 3 Department of Biology, University of Utah, Salt Lake City, UT 84112, USA; maren.watkins@hsc.utah.edu (M.W.); s.robinson@imb.uq.edu.au (S.D.R.); 4 Department of Biochemistry, University of Utah, Salt Lake City, UT 84112, USA; 5 USTAR Center for Genetic Discovery, University of Utah, Salt Lake City, UT 84112, USA

**Keywords:** machine learning, conotoxins, cone snails, venom, drug discovery

## Abstract

Cone snails (genus *Conus*) are venomous marine snails that inject prey with a lethal cocktail of conotoxins, small, secreted, and cysteine-rich peptides. Given the diversity and often high affinity for their molecular targets, consisting of ion channels, receptors or transporters, many conotoxins have become invaluable pharmacological probes, drug leads, and therapeutics. Transcriptome sequencing of *Conus* venom glands followed by de novo assembly and homology-based toxin identification and annotation is currently the state-of-the-art for discovery of new conotoxins. However, homology-based search techniques, by definition, can only detect novel toxins that are homologous to previously reported conotoxins. To overcome these obstacles for discovery, we have created *Conus*Pipe, a machine learning tool that utilizes prominent chemical characters of conotoxins to predict whether a certain transcript in a *Conus* transcriptome, which has no otherwise detectable homologs in current reference databases, is a putative conotoxin. By using *Conus*Pipe on RNASeq data of 10 species, we report 5148 new putative conotoxin transcripts that have no homologues in current reference databases. 896 of these were identified by at least three out of four models used. These data significantly expand current publicly available conotoxin datasets and our approach provides a new computational avenue for the discovery of novel toxin families.

## 1. Introduction

Predatory marine cone snails (genus *Conus*) have attracted the attention of biologists and pharmacologists for the great neuropharmacological potential of their venom toxins [1,2,3]. It is estimated that each of the ~750 extant *Conus* species produces ~100–400 distinct venom toxins (conotoxins) with almost no overlap in the toxin repertoire between the ~750 species, not even between sister species [4]. Despite the tremendous diversity and drug discovery potential of *Conus* venoms, only ~5000 nucleotide sequences of conotoxin-encoding transcripts have been reported from 100 *Conus* species over the past decades, with most sequences having been discovered in recent years [5,6]. Traditional methods, such as isolation of conotoxins from venom and subsequent Edman- or de novo mass spectrometric (MS) sequencing are time-consuming and limited by sample availability. In contrast, high throughput transcriptome sequencing can achieve greater sequencing depth and only requires small amounts of biological sample [7]. Recent studies on the venom gland transcriptomes of several cone snail species, using next generation sequencing technologies (NGS), have discovered ~100–400 conotoxin genes per *Conus* species [4,8,9,10,11,12]. Other authors have reported larger diversities but these are likely to have resulted from inappropriate analyses of NGS datasets, as previously discussed [4,10].

Conotoxins can be classified into different gene superfamilies based on their conserved N-terminal signal sequence [13]. To date, more than 53 conotoxin gene superfamilies have been described for *Conus* [14]. After NGS sequencing and *de novo* transcriptome assembly, candidate conotoxin genes are usually assigned to different superfamilies using BlastX, regular expression-based techniques, and profile hidden Markov model (HMMER) analysis against a local reference database of known conotoxins from the Uniprot and/or ConoServer databases. Available tools include ConoPrec, ConoDictor, and Conosorter [5,15,16,17]. However, these approaches can only detect novel toxins that are similar to previously reported sequences. An approach that could overcome the limitations of homology based-searches would thus be highly desirable.

Most conotoxin transcripts can be readily divided into three distinct regions: (1) an N-terminal signal sequence for targeting to the endoplasmic reticulum; (2) an intermediate propeptide region that has been suggested to play a role in secretion, posttranslational modification, and folding; and (3) a single copy of the mature toxin region, located at the C terminus [18,19,20]. We hypothesized that even though conotoxin sequences evolve very rapidly [14], conotoxin retain these three traits even when their sequence similarities become too low to allow detection by alignment-based methods such as Blast and HMMER. If this were true, even highly divergent conotoxins might be identifiable using machine learning methods trained to identify these three traits as features. With this in mind, we implemented three machine learning models for data mining of 12 *Conus* transcriptomes from 10 different species: logistic regression (logit), semi-supervised learning (labelspreading), and an artificial neural network (perceptron) [21,22,23,24,25]. The resulting tool for conotoxin discovery is called *Conus*Pipe.

Generalized linear models (GLMs) are versatile, powerful, commonly used statistical approaches to model relationship between scalar response variables given several predictor variables/features [21,26]. In particular, the logistic regression model computes a weighted sum of the input features (plus a bias term), but instead of outputting the result directly like the linear regression model does, it outputs the logistic of this result [21,25]. This approach often outperforms simple linear regression for binary outcome prediction [26].

Unlike GLMs which completely employ ‘labeled data’ for training, in semi-supervised learning only some of the training data is labeled. In the models utilized in the current study, the term ‘labeled data’ refers to known training sequences labeled as either toxin or non-toxin. Unlabeled data refers to sequences of unknown category. Graph-based methods are then used to make use of unlabeled data in order to better capture the shape of the underlying data distribution and generalize the method to apply to new samples [27]. The assumption is that unlabeled data with features that render them neighbors of labeled data are likely to have a common label. In keeping with general practice, we used the K-nearest neighbors method to connect each data point [28]. Semi-supervised learning approaches can perform well when only a small number of labeled data but large amounts of unlabeled data is available.

The perceptron is one of the simplest artificial neural network ANN architectures, which is composed of a single layer of linear threshold unit (LTU). The LTU computes a weighted sum of its inputs and then applies a step function to that sum and outputs the result [22]. Unlike regression-based approaches, ANNs can capture dependencies within the data, potentially resulting more accurate classification.

The nature of the *Conus* training sets and input data also needs to be considered. For example, the true-positive (labeled) training data is limited in scale and is incomplete, as many conotoxins presumably remain uncatalogued [29]. Moreover, the input data is very large, with RNA-seq datasets typically exceeding five million reads and tens of thousands of assembled transcripts. Given these features, logistic regression provides a well-established base-line approach, whereas semi-supervised learning (labelspreading) provides a means to further leverage unlabeled true positives during training. Finally, the Perceptron model scales well to very large training sets and is widely used for pattern recognition with good results [30,31,32,33]. Both the logit and perceptron models are supervised and model-based, in contrast, the label spreading model is semi-supervised and instance-based. Given these machine learning models have intrinsic strengths and weaknesses and are complementary to one another, using an ensemble of these methods is likely to provide best discovery outcomes. Additionally, since using these machine learning models are based on the hypothesis that conotoxins will retain all three traits in sequence evolution, we added cross-species Blastp to search for similar unknown sequences (if they contain a signal sequence) between different *Conus* species to rescue potential conotoxins that only have one trait (signal sequence) based on the knowledge that the signal peptide sequences of conotoxins from the same superfamilies are highly similar to each other even if they are from different *Conus* species [4].

By employing three different machine learning models plus Blastp, *Conus*Pipe allows users to take an ensemble approach for discovery to maximize the prediction power. All four methods were applied to 12 RNAseq datasets from 10 different species of *Conus*. The pipeline discovered 5148 new conotoxin candidates that provide a unique dataset for future pharmacotherapeutic exploration.

## 2. Results

### 2.1. The ConusPipe Toolkit

**Figure 1:**
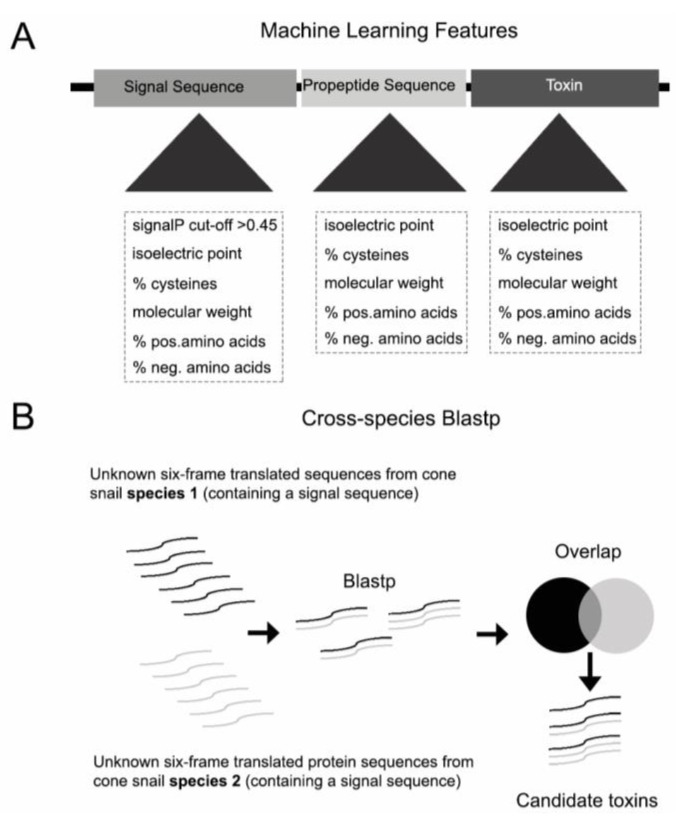
(**A**) Overview of feature selection for machine learning models (**B**) and cross-species Blastp methodology used in addition to the machine learning model.

*Conus*Pipe is implemented in Perl and Python as a complete conotoxin discovery package. It is available at https://github.com/Yandell-Lab/ConusPipe. *Conus*Pipe takes six-frame-translated peptide sequences from nucleotide sequences which have no hit in current reference database and extracts conotoxin sequence features to train datasets (Figure 1A). In addition to three machine learning models, cross-species Blastp is used as the fourth method to retrieve putative toxin candidates that have a signal sequence but may not have all features used in machine learning (Figure 1B).

**Figure 2:**
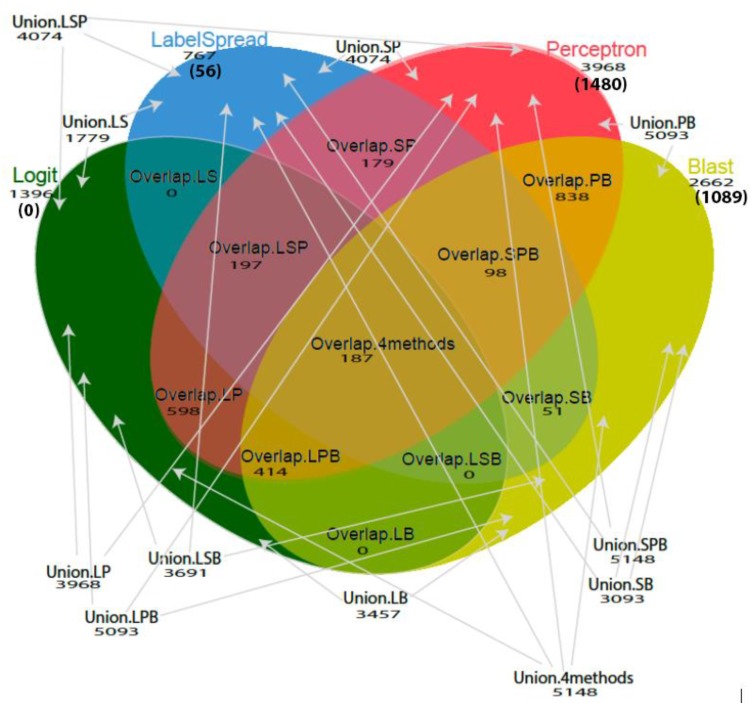
Venn diagram illustrates the different combinations of methodologies used (single method, overlap, or union of methods) and the total number of putative toxin candidates identified by each method (unique number of toxin candidates are shown in parentheses). A union of methods means that a conotoxin is predicted by one or more methods, for example, Union.4methods = predicted by perceptron or logit or label spreading or blast. An overlap of methods means that the conotoxin is predicted by all the applied methods, for example, Overlap.4methods = predicted by perceptron and logit and label spreading and blast. Abbreviations used: blast—B; logit—L; labelspreading—S; perceptron—P.

*Conus*Pipe then generates different combinations (single method, union or overlap) of the four methods to predict candidate conotoxins (Figure 2). Users can change the settings in sample.config file to use different cut-off options for transcript per million (tpm) values, blast e-values, signalP D-values, and provide paths to input and output fasta files and databases used. It took 2 h 34 min 20 s on a single CPU core to run *ConusPipe* for 757,932 *Conus* transcripts from 12 samples of 10 species.

### 2.2. Building and Cross-Validation of the Machine Learning Models

4950 known conotoxin sequences (from the ConoServer [5] and Uniprot databases [34], and 52,613 randomly selected non-conotoxin *Conus* transcripts were used to build the machine learning models. In order to assess the performance of the models, 10-fold cross validation was applied to the same dataset [35]. The main measures of performance were sensitivity, specificity and accuracy under different regularization parameters. Sensitivity was defined as the fraction of known conotoxins predicted as conotoxin divided by the number of known conotoxins in the test dataset. Specificity was defined as the fraction of known non-conotoxins predicted as non-conotoxin divided by the number of known non-conotoxins in the test dataset. Accuracy was defined as the fraction of known sequences (conotoxin/non-conotoxin) predicted divided by the total number of sequences in the test dataset. The regularized parameter settings were chosen by plotting accuracy vs. parameter settings for each model to make sure the trained model has the best accuracy with minimum overfitting/under fitting. Since the prevalence of conotoxins in the training dataset is only 9.97%, sensitivity is an important performance measure in consideration when choosing regularization parameter settings. The sensitivity, specificity, and accuracy for the chosen regularization parameter settings for each model is shown in Table 1. The highest overall testing accuracy and sensitivity were 98.2% and 90.93%, respectively, achieved by the label spreading model.

### 2.3. Benchmark by Identifying Known Superfamilies

To assess the sensitivity of *Conus*Pipe in identifying conotoxin transcripts, we performed a benchmark analysis for sequences belonging to known conotoxin gene superfamilies [6]. For these analyses, we deleted an entire superfamily from the training set and then tried to re-discover this superfamily as a putative new superfamily using *Conus*Pipe. The specificity of *Conus*Pipe (i.e., the ability to distinguish between known conotoxins and non-conotoxin proteins from various organisms) was assessed by screening hits against the entire UniProtKB/Swiss-Prot database.

We found that the sensitivity varied among different superfamilies and combinations of models used. The highest sensitivity was achieved by the union of the four methods (logit, labelspreading, perceptron and blastp, mean sensitivity = 95.7%, SD = 0.11) and the union of three methods (logit, perceptron and blastp or labelspreading, perceptron and Blastp, mean sensitivity = 95.7%, SD = 0.11) across all superfamilies. A union of methods means that a conotoxin predicted by one or more methods is a putative positive. A graphical overview of the different groups is provided in Figure 2. Results are shown in Figure S1 and Table 1 and Table 2.

In addition to machine learning, using cross-species Blastp to search candidate sequences from different *Conus* species against each other provides better performance for conotoxin superfamilies that contain sequences which do not satisfy the hypothesis of having all three traits (signal sequence, propeptide, and mature toxin at the C-terminus), such as the SF-mi2 (Superfamily-2 *Conus miles*), I4, B4 and Prohormone gene families. However, this approach is less powerful for superfamilies that are limited to a small number of species, such as conorfamides, DivMTFLLLLVSV (Diverse MTFLLLLVSV), teretoxins, and conocaps. The sensitivity for recovering all known conotoxin superfamilies by different combinations of methods is provided in Supplementary Table 1.

The ability of *Conus*Pipe to distinguish between conotoxins and other proteins from various organisms was assessed by screening the entire UniProtKB/Swiss-Prot database. Using the version released on June 2013 we examined a total of 540,261 protein sequences isolated from diverse organisms. The overall highest specificity was achieved using an overlap of the four methods (logit, labelspreading, perceptron, and blastp, specificity = 99.92%) and the overlap of three methods (labelspreading, perceptron, and blastp, specificity = 99.92%). These results are shown in Table 2.

### 2.4. Identification of New Conotoxin Candidates

To identify new conotoxin candidates, we assembled *Conus* transcripts from RNA-seq datasets derived from venom glands of 10 different *Conus* species using our previously published methods [4] (see Table 3 for species used in this study). The resulting transcripts were prescreened for conotoxin homology against the Uniprot and ConoServer databases as previously published [4] and described under the methods section. The remaining transcripts were then used as inputs to *Conus*Pipe. New conotoxin candidates were defined as those which were predicted as conotoxins by at least one of the four models in *Conus*Pipe, but lacked significant homology to known conotoxins using Blast against the Uniprot/ConoServer database [17].

Since conotoxin gene superfamilies are generally found across multiple *Conus* species (hence, the term superfamily) we considered those sequences that lacked significant homology to known conotoxins but had high homology (blastp e-value < 1 × 10^−10^ to sequences from at least two other *Conus* species examined here, as members of a new putative superfamily. 5479 transcripts passed these criteria. In order to validate our predictions, we used NCBI-Blastp to search the 5479 transcripts against the NCBI non-redundant (NR) protein database (August 2018 Version), which includes recently published conotoxin sequences that were not yet available in Uniprot/ConoServer at the time of original analysis (and even now) and also includes large numbers of uncharacterized molluscan sequences not available in Uniprot. Out of 5479 transcripts, 331 had significant blastp hits (e-values < 1 × 10^−4^) against the NCBI-NR database. 99 transcripts had hits against other molluscan transcripts. As the majority of conotoxins are not found outside of the genus *Conus* and these transcripts could encode endogenous signaling/housekeeping polypeptides rather than polypeptides used for envenomation, these were removed from our final datasets. 198 sequences were identified as conotoxins. These were also removed from the final machine learning dataset and are provided in Supplementary File 1. Finally, 34 sequences had blastp hits against non-molluscan species such as fish, tardigrade, sea anemone, worm, plant, and bird. These were removed from the final dataset.

A total of 5148 sequences were left in our final dataset as new conotoxin candidates, 187 of these were identified by all four models, 709 of these were identified by three models, 1666 of these were identified by two models, 2586 of these were identified by one model (see Figure 1 for toxin candidates identified by each model and their overlap and unions). The logit model did not uniquely identify any toxin candidates; all its 1396 newly discovered putative toxins are overlapped with other models. The labelspreading model, perceptron model, and blastp uniquely discovered 56, 1480, and 1089 new potential toxins, respectively. These results highlight that using an ensemble of different models takes advantage of the complementary aspects of each model to maximize discovery. All of the 5148 transcripts are provided in Supplementary File 2. Using single linkage cluster analysis with a Jaccard Index of 0.5 [36,37] grouped 299 of sequences identified by at least three models into 114 clusters that are likely to represent novel conotoxin gene superfamilies (Supplementary File 3).

The highest number of putative new sequences were identified in *C. striatus*. Blastp against Uniprot/ConoServer identified 34 toxin transcripts in *C. striatus* and 25 of these were also retrieved using the machine learning method (i.e., 9 of these were missed by machine learning, see discussion section below). 1079 additional putative toxins were subsequently identified by machine learning, 10 of these were confirmed as toxins by Blastp against the NCBI-NR database (Table 3). All toxins identified per species are provided in Supplementary File 4 (“sp.all.tar.gz”), including toxins with Blast hits against NCBI/Uniprot/ConoServer and toxin candidates identified by *Conus*Pipe.

### 2.5. Transcripts Identified Using Three or Four out of Four Methods

**Figure 3:**
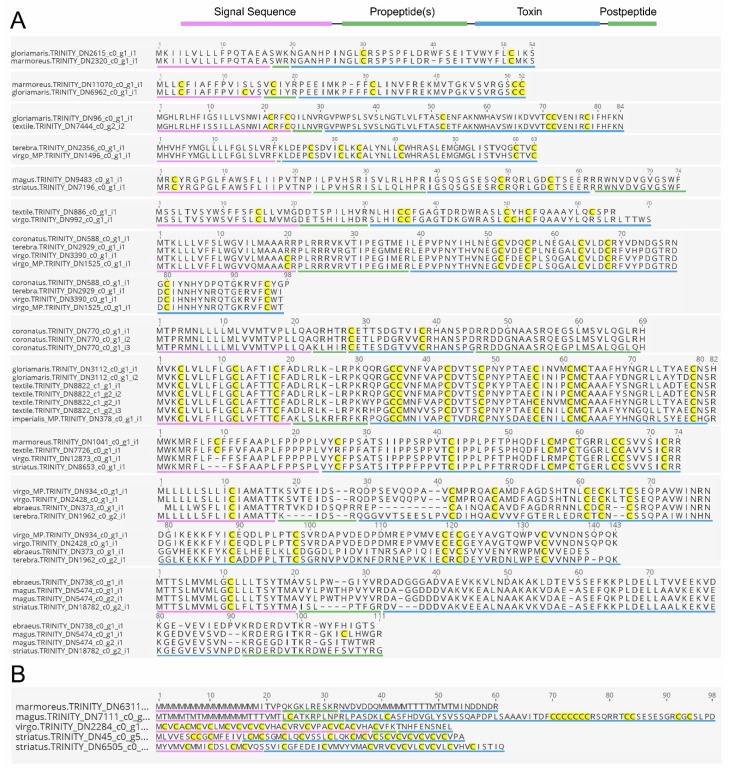
Comparative alignments of selected sequences identified by at least three out of four methods that are (**A**) likely or (**B**) not likely to represent genuine novel conotoxins. Cysteines are highlighted in yellow, signal sequences, pro- and postpeptides and predicted mature toxins are underlined in purple, green, and blue, respectively, as shown on top of panel A. Sequence labels (contigs) correspond to those provided in Supplementary File 2.

In this study, we provide a list of all new conotoxin candidates from the venom gland transcriptomes of several cone snail species. We emphasize that these sequences require further experimental validation to confirm their designation as genuine conotoxins. This is discussed in more detail below. However, we propose that many of the transcripts identified by three out of four methods (709 sequences) or by a combination of all four methods (187 sequences) are likely to represent genuine conotoxin sequences since they were independently identified using different approaches. Several of these sequences exhibit clear hallmarks of conotoxins (presence of propeptides, found in multiple species, similar but distinct sequences found in different species, multiple cysteines in mature toxin region). An alignment of some of these is shown in Figure 3. Several sequences that were identified by three or more models but seem unlikely to represent genuine conotoxins are also shown. These typically exhibit a long series of cysteine repeats, several vicinal cysteines, and/or series of methionines (M) in the signal sequence. In the future, our model could be further refined to exclude such sequences as candidates.

## 3. Discussion

Previous approaches for toxin discovery have been alignment based, using regular expressions, blast, and/or HMMER to identify new members of known conotoxin superfamilies [5,15,16,17]. Because conotoxins are hyperdiverse, these approaches are intrinsically limited. In an attempt to cast a wider net for discovery, we have created a machine learning based pipeline, *Conus*Pipe, that utilizes functional characteristics of conotoxins to identify new conotoxin candidates that have no significant sequence homology to conotoxin sequences currently available in reference databases.

By using more than one machine learning model, we expected to see that an ensemble of different models can maximize the prediction power. Indeed, as determined by benchmark analyses, the highest sensitivity is achieved by the union of three or more methods and the highest specificity is achieved in the overlap of three or more methods.

*Conus*Pipe allows users to choose different combinations of methods according to their requirement on discovery specificity and sensitivity (Table 1). In addition to developing machine learning, we demonstrate that using blast to search candidate sequences from different *Conus* species against each other provides better performance for conotoxin superfamilies that contain sequences that do not satisfy the hypothesis of having all three traits. However, this approach is less powerful for superfamilies that are limited to a small number of species and only works if more than one transcriptome is to be analyzed.

We would like to note that, for best performance, *Conus*Pipe should be used in combination with currently used homology-based search algorithm, such as blast, as *Conus*Pipe relies on the presence of full-length sequences (containing N-terminal signal sequences) and will not work for truncated contigs. Furthermore, several conotoxin gene superfamilies reported in Uniprot/ConoServer have low SignalP values (D value of <0.45). These would also be missed by our pipeline. Table 3 provides information on how many conotoxins which have significant homology to sequences in Uniprot/ConoServer database could also be retrieved by *Conus*Pipe (average value for recovery: 65%, ranging from 57% for *C. virgo* to 82% for *C. imperialis*).

We provide a large set of new conotoxin candidates and a bioinformatic pipeline that is freely available and can be applied to any newly sequenced *Conus* venom gland. No doubt our approach also identifies false positives, particularly when only a single or a combination of two methods is employed. Thus, using our method without further validation is not suitable for defining the venom composition of a species. What it provides is a new tool to identify candidate toxin transcripts that are not able to be detected by homology-based methods. Generating comprehensive databases of all putative toxin candidates expressed in a venom gland will empower current mass spectrometric toxin sequencing approaches [7]. Using mass spectrometry, candidate transcripts can then be verified and subjected to functional characterizations.

## 4. Conclusions

Using *Conus*Pipe, we identified 5148 new conotoxin candidates from 757,932 transcripts derived from venom gland transcriptomes of 10 *Conus* species. None of these candidate conotoxins has significant homology to any known conotoxin in the Uniprot/ConoServer database, although like known conotoxins, most candidates have an N-terminal signal sequence, a characteristic propeptide spacer region, and a single copy of a mature peptide at the C-terminus. Moreover, we have shown that several of these candidates share high homology to newly published conotoxins in the NCBI-NR molluscan database. In conclusion, our approach opens new avenues for the discovery of novel conotoxin transcripts from cone snails and other venomous animals with similar venom repertoires.

## 5. Materials and Methods

### 5.1. Transcriptome Sequencing

Specimen were collected in the central Philippines during several collection expeditions in 2011–2015. Specimen identification was initially performed by morphological examination and later verified by sequence analysis of the cytochrome oxidase c subunit 1 (COI) gene. Venom glands were dissected and stored in RNAlater at −80 °C until further processing. Total RNA was isolated from venom glands using *TRIzol*^®^ *Reagent* (Invitrogen, ThermoFisher Scientific, Waltham, MA, USA) or the RNeasy kit (Qiagen, Germantown, MD, USA) following the manufacturers’ instructions. RNA integrity, quantity, and purity were determined on a 2100 Bioanalyzer (Agilent Technologies, Santa Clara, CA, USA). cDNA libraries were prepared and sequenced on an Illumina HiSeq 2000 instrument (Sanger/Illumina 1.9 reads, 101 bp or 125 bp paired-end, Illumina, San Diego, CA, USA). Publicly available Illumina datasets were used for the venom gland transcriptomes of *C. marmoreus* (specimen 2), *C. virgo* (specimen 2), *C. coronatus*, and *C. ebraeus* [10].

Adapter clipping and quality trimming of raw reads were performed using fqtrim software (Version 0.9.4, http://ccb.jhu.edu/software/fqtrim/) and PRINSEQ (Version 0.20.4 [38]). After processing, sequences shorter than 70 bps and those containing more than 5% ambiguous bases (Ns) were discarded. De novo transcriptome assembly was performed using Trinity Version 2.0.5 [39] with a kmer size for building De Bruijn Graphs of 31, a minimum kmer coverage of 10, and a minimum glue of 10. Assembled transcripts were annotated using Blastx ((NCBI-Blast-2.2.28+, [40]) against conotoxin sequences extracted from the ConoServer [5] and UniProt databases [34].

### 5.2. Development of ConusPipe

*Conus*Pipe proceeds by first extracting 16 features (see feature explanation below) from known conotoxin sequences (from the ConoServer [6] and Uniprot databases) and non-conotoxin *Conus* transcripts to make the training dataset. Sequences are required to contain a signal sequence as determined by SignalP [41]. Next, the 16 features extracted from *Conus* transcripts of 10 species, which have no homologues in current reference database (the combined ConoServer and UniProtKB database) are used as real test data to run the machine learning methods to predict whether a certain input transcript is conotoxin. All the transcripts predicted by any of the four methods are output by the pipeline as putative new conotoxins.

#### 5.2.1. Feature Selection and Classifiers

We employed 16 features for the machine learning models: signalP D value for signal sequence; cysteine percentage; molecular weight; percentage of positively/negatively charged amino acids; and isoelectric point for all three regions of the precursor sequence (signal sequence, propeptide, and mature toxin). All features are continuous re-normalized to lie between 0 and 1 (Figure 1A). The features are mainly chemical characters of amino acids in the three different parts of conotoxin sequence. The motivating hypothesis is that even though conotoxins evolve very rapidly they must still share similar chemical characters in amino acid composition, since they carry out similar functions, e.g., bind transporters and receptors. For example, the three parts of conotoxin sequence-signal sequence, propeptide, and mature toxin carry out different functions in conotoxin secretion process in the cell, so their charge distributions are stereotypical and different. The signal sequence is mainly hydrophobic, while the amino acids in propeptide are mainly charged, and the mature toxin is somewhat intermediate as regards charge distribution. To train the models we first ran signalP on a training dataset consisting of known conotoxin/nonconotoxin sequences to get the signalP D value for each known sequence [41]. Next, we calculated the cysteine percentage, molecular weight, percentage of positively/negatively charged amino acids and isoelectric point for signal sequence, propeptide and mature toxin, respectively. The pipeline uses the 16 extracted features in training dataset to train the logistic regression model, labelspreading model, and perceptron model using the Python scikit learn package [42]. The accuracy under different regularization parameters of the three models were tested by cross validation with training dataset from known conotoxin and nonconotoxin sequences.

#### 5.2.2. Cross Validation

4950 known conotoxin sequences (from ConoServer and Uniprot/Swissprot) and 52,613 non-conotoxin *Conus* transcripts with matched sequence length were first split into 10 equal bins, and then sequentially one-tenth of the data was taken as the test set and the remaining other nine-tenths were used for the training set. The three models were trained under different regularization parameters and evaluated with the test set in 10 iterations. For the logistic regression model, the regularization parameter we tested is slack number C, which is the inverse of regularization strength. We tested this parameter from 0.001 to 1 × 10^10^. For the labelspreading model, we chose knn as the kernel function, so the regularization parameter we tested is n_neighbors, which was tested from 1 to 15. For the perceptron model, the regularization parameter we tested is n_iter, the number of passes over the training data, which we tested from 5 to 70. The plots of accuracy versus regularization parameter of different models are shown in Figure S2.

#### 5.2.3. Discovering New Putative Conotoxins and Conotoxin Gene Families

Paired-end RNAseq data from 10 *Conus* species were generated by Illumina HiSeq 2000 platform (Table 4). RNAseq reads were assembled using best practice Trinity settings, annotated with Blastx against our reference dataset, and all the *Conus* transcripts which do not have homologous sequences in the current reference databases were selected and six-frame-translated into peptide sequences. These peptide sequences were then used as the input dataset for *Conus*Pipe to drive machine learning models built in previous steps to predict whether they are conotoxins. Cross-species Blastp is also used on all putative peptide sequences that have a signal sequence as an independent method to predict putative conotoxin candidates. The pipeline output all input transcripts which were predicted as conotoxins.

We then used NCBI-Blastp to search all putative toxin transcripts against the NCBI non-redundant (NR) protein database (August 2018 version), which includes recently published conotoxin sequences that were not yet available in Uniprot/ConoServer at the time of original analysis. Sequences with significant Blastp hits were excluded from final datasets (e-values < 1 × 10^−4^).

Then all by all Blastp and single linkage cluster analysis were conducted among the new conotoxins, and the new conotoxins that shared at least 50% hit connections with one another were designated as to be in the same superfamily.

## Figures and Tables

**Table 1:** Maximized sensitivity, specificity, and accuracy for chosen regularization parameter settings for three machine learning models in 10-fold cross validation.

Machine Learning Model	Performance Measure		
	Sensitivity	Specificity	Accuracy
Logit	82.85%	99.30%	97.78%
Label spreading	90.93%	99.07%	98.32%
Perceptron	83.24%	97.65%	96.32%

**Table 2:** Specificity of the individual machine learning methods and their unions/combinations when searching results against the Uniprot/Swissprot non-conotoxin database and mean sensitivity for recovering all known conotoxin superfamilies. Methods are ordered as follows: Overlap between different methods, single methods, and union of methods. Methods with the highest sensitivities (≥99.7%) and specificities (≥99.9%) are shown in bold. Abbreviations used: blast—B; logit—L; label spreading—S; perceptron—P.

Methods	Mean Sensitivity	Specificity
Overlap.4methods	34.19% ± 0.32%	**99.92%**
Overlap.LSP	41.53% ± 0.35%	**99.90%**
Overlap.LSB	35.15% ± 0.32%	99.87%
Overlap.LPB	76.25% ± 0.37%	99.57%
Overlap.SPB	34.22% ± 0.32%	**99.92%**
Overlap.LS	43.18% ± 0.34%	99.85%
Overlap.LP	83.61% ± 0.32%	99.53%
Overlap.SP	41.68% ± 0.35%	**99.90%**
Overlap.LB	79.57% ± 0.35%	98.32%
Overlap.SB	35.52% ± 0.32%	99.86%
Overlap.PB	78.02% ± 0.36%	99.57%
Logit	87.61% ± 0.26%	99.83%
Labelspreading (SemiS)	43.67% ± 0.34%	99.49%
Perceptron (NeuroNetWork)	85.96% ± 0.29%	94.02%
Blastp	87.10% ± 0.28%	98.19%
Union.4methods	**95.73% ± 0.11%**	93.89%
Union.LSP	90.31% ± 0.22%	98.15%
Union.LSB	95.25% ± 0.12%	93.89%
Union.LPB	**95.73% ± 0.11%**	93.90%
Union.SPB	**95.73% ± 0.11%**	93.97%
Union.LS	88.10% ± 0.26%	98.17%
Union.LP	89.96% ± 0.23%	98.17%
Union.SP	87.95% ± 0.25%	99.41%
Union.LB	95.14% ± 0.12%	93.90%
Union.SB	95.24% ± 0.12%	93.99%
Union.PB	95.05% ± 0.15%	93.98%

**Table 3:** Conotoxin candidates expressed in 12 samples from 10 *Conus* species identified by blastp and machine learning (ML).

*Conus* Species	No. of Conotoxins Identified by Blastp against Uniprot/ConoServer Database	No. of Conotoxins Identified by Blastp against Uniprot/ConoServer Database Also Retrieved Using ML	No. of Conotoxins Identified by ML and Subsequently Identified as Conotoxins by Blastp against NCBI	No. of Conotoxin Candidates Identified by ML Only
*C. magu*	49	29	13	984
*C. striatus*	34	25	10	1069
*C. marmoreus*	21	13	9	522
*C. marmoreus2* [10]	16	10	3	61
*C. textile*	95	58	19	532
*C. gloriamaris*	62	38	15	529
*C. imperialis* [10]	29	24	17	61
*C. virgo*	54	31	23	739
*C. virgo2* [10]	37	22	6	44
*C. terebra*	67	46	21	389
*C. coronatus* [10]	94	59	38	97
*C. ebraeus* [10]	17	13	24	121

**Table 4:** RNAseq Data sets from 12 samples in 10 *Conus* species used in the discovery pipeline.

*Conus* Species	Illumina HiSeq 2000		SRA Accession Number
	Number of Reads	Read Length (nt)	
*C. magus*	85,877,500	101	SRX5015024
*C. striatus*	101,170,402	101	SRX5015022
*C. marmoreus*	53,901,510	125	SRX5015020
*C. marmoreus2* [10]	50,652,396	101	SRX1323884
*C. textile*	63,365,620	125	SRX5015023
*C. gloriamaris*	28,783,428	125	SRX2779517
*C. imperialis* [10]	30,784,548	125	SRX1323891
*C. virgo*	30,038,902	125	SRX5015021
*C. virgo2* [10]	31,056,732	125	SRX1323883
*C. terebra*	31,180,460	125	SRX5015025
*C. coronatus* [10]	27,927,952	125	SRX1323894
*C. ebraeus* [10]	19,556,244	125	SRX1323887

## Supplementary Materials

The following are available online at http://www.mdpi.com/2072-6651/10/12/503/s1, Figure S1: Box plot shows that the sensitivity varied among different combinations (single method, overlap or union of methods) of methods used. Figure S2: Plot of accuracy vs. regularization parameter settings in each machine learning model. Supplementary Table 1. The sensitivity for recovering all known conotoxin superfamilies by different combinations of methods. Supplementary File 1. *Conus*Pipe discovered 198 putative novel conotoxins confirmed by Blastp against the NCBI NR database. Supplementary File 2. *Conus*Pipe discovered 5148 putative novel conotoxins with no significant sequence homology in any current reference database (Unipro/ConoServer/NCBI NR). Supplementary File 3. Cluster analysis of putative new gene superfamilies. Supplementary File 4 (“sp.all.tar.gz”). All sequences identified for each *Conus* species in this study.

## References

[1] Shen G.S., Layer R.T., McCabe R.T. (2000). Conopeptides: From deadly venoms to novel therapeutics. Drug Discov. Today.

[2] McIntosh J.M., Jones R.M. (2001). Cone venom—From accidental stings to deliberate injection. Toxicon.

[3] Livett B.G., Gayler K.R., Khalil Z. (2004). Drugs from the sea: Conopeptides as potential therapeutics. Curr. Med. Chem..

[4] Li Q., Barghi N., Lu A.P., Fedosov A.E., Bandyopadhyay P.K., Lluisma A.O., Concepcion G.P., Yandell M., Olivera B.M., Safavi-Hemami H. (2017). Divergence of the venom exogene repertoire in two sister species of *Turriconus*. Genom. Biol. Evol..

[5] Kaas Q., Westermann J.C., Halai R., Wang C.K., Craik D.J. (2008). ConoServer, a database for conopeptide sequences and structures. Bioinformatics.

[6] Kaas Q., Westermann J.C., Craik D.J. (2010). Conopeptide characterization and classifications: An analysis using ConoServer. Toxicon.

[7] Robinson S.D., Undheim E.A.B., Ueberheide B., King G.F. (2017). Venom peptides as therapeutics: Advances, challenges and the future of venom-peptide discovery. Expert Rev. Proteom..

[8] Robinson S.D., Safavi-Hemami H., McIntosh L.D., Purcell A.W., Norton R.S., Papenfuss A.T. (2014). Diversity of conotoxin gene superfamilies in the venomous snail, *Conus victoriae*. PLoS ONE.

[9] Robinson S.D., Li Q., Lu A., Bandyopadhyay P.K., Yandell M., Olivera B.M., Safavi-Hemami H. (2017). The Venom Repertoire of *Conus gloriamaris* (Chemnitz, 1777), the Glory of the Sea. Mar. Drugs.

[10] Phuong M.A., Mahardika G.N., Alfaro M.E. (2016). Dietary breadth is positively correlated with venom complexity in cone snails. BMC Genom..

[11] Hu H., Bandyopadhyay P.K., Olivera B.M., Yandell M. (2012). Elucidation of the molecular envenomation strategy of the cone snail *Conus geographus* through transcriptome sequencing of its venom duct. BMC Genom..

[12] Barghi N., Concepcion G.P., Olivera B.M., Lluisma A.O. (2015). Comparison of the venom peptides and their expression in closely related *Conus* species: Insights into adaptive post-speciation evolution of *Conus* exogenomes. Genom. Biol. Evol..

[13] Buczek O., Bulaj G., Olivera B.M. (2005). Conotoxins and the posttranslational modification of secreted gene products. Cell Mol. Life Sci..

[14] Olivera B.M., Safavi-Hemami H., Horvarth M.P., Teichert R.W., Baker B.J. (2015). Conopeptides, Marine Natural Products from Venoms: Biomedical Applications and Future Research Applications. Marine Biomedicine: From Beach to Bedside.

[15] Koua D., Brauer A., Laht S., Kaplinski L., Favreau P., Remm M., Lisacek F., Stocklin R. (2012). ConoDictor: A tool for prediction of conopeptide superfamilies. Nucleic Acids Res..

[16] Lavergne V., Dutertre S., Jin A.H., Lewis R.J., Taft R.J., Alewood P.F. (2013). Systematic interrogation of the *Conus marmoreus* venom duct transcriptome with ConoSorter reveals 158 novel conotoxins and 13 new gene superfamilies. BMC Genom..

[17] Wheeler T.J., Eddy S.R. (2013). nhmmer: DNA homology search with profile HMMs. Bioinformatics.

[18] Bandyopadhyay P.K., Colledge C.J., Walker C.S., Zhou L.M., Hillyard D.R., Olivera B.M. (1998). Conantokin-G precursor and its role in gamma-carboxylation by a vitamin K-dependent carboxylase from a *Conus* snail. J. Biol. Chem..

[19] Conticello S.G., Kowalsman N.D., Jacobsen C., Yudkovsky G., Sato K., Elazar Z., Petersen C.M., Aronheim A., Fainzilber M. (2003). The prodomain of a secreted hydrophobic mini-protein facilitates its export from the endoplasmic reticulum by hitchhiking on sorting receptors. J. Biol. Chem..

[20] Buczek O., Olivera B.M., Bulaj G. (2004). Propeptide does not act as an intramolecular chaperone but facilitates protein disulfide isomerase-assisted folding of a conotoxin precursor. Biochemistry.

[21] Cox D.R. (1958). The Regression-Analysis of Binary Sequences. J. R. Stat. Soc. B.

[22] Pollack J.B. (1989). Perceptrons—An Introduction to Computational Geometry, Expanded Edition—Minsky, Ml, Papert, Sa. J. Math. Psychol..

[23] Widrow B., Lehr M.A. (1990). 30 Years of Adaptive Neural Networks-Perceptron, Madaline, and Backpropagation. Proc. IEEE.

[24] Delalleau O., Bengio Y., Le Roux N. (2005). Efficient Non-Parametric Function Induction in Semi-Supervised Learning. AISTATS.

[25] Yu H.F., Huang F.L., Lin C.J. (2011). Dual coordinate descent methods for logistic regression and maximum entropy models. Mach. Learn..

[26] Zhao L., Chen Y., Schaffner D.W. (2001). Comparison of logistic regression and linear regression in modeling percentage data. Appl. Environ. Microbiol..

[27] Belkin M., Niyogi P. (2004). Semi-supervised learning on Riemannian manifolds. Mach. Learn..

[28] Altman N.S. (1992). An Introduction to Kernel and Nearest-Neighbor Nonparametric Regression. Am. Stat..

[29] Robinson S.D., Norton R.S. (2014). Conotoxin gene superfamilies. Mar. Drugs.

[30] LeCun Y., Boser B., Denker J.S., Henderson D., Howard R.E., Hubbard W., Jackel L.D. (1989). Backpropagation Applied to Handwritten Zip Code Recognition. Neural Comput..

[31] Hyvarinen A., Koster U. (2007). Complex cell pooling and the statistics of natural images. Network-Comp. Neural.

[32] He K.M., Zhang X.Y., Ren S.Q., Sun J. (2015). Spatial Pyramid Pooling in Deep Convolutional Networks for Visual Recognition. IEEE Trans. Pattern Anal..

[33] Zhou L.J., Li Q.W., Huo G.Y., Zhou Y. (2017). Image Classification Using Biomimetic Pattern Recognition with Convolutional Neural Networks Features. Comput. Intell. Neurosci..

[34] Consortium U. (2015). UniProt: A hub for protein information. Nucleic Acids Res..

[35] Picard R.R., Cook R.D. (1984). Cross-Validation of Regression-Models. J. Am. Stat. Assoc..

[36] Tan P.N., Steinbach M., Kumar V. (2005). Introduction to Data Mining.

[37] Jaccard P. (1901). Distribution de la flore alpine dans le Bassin des Dranses et dans quelques regions voisines. Bull. Soc. Vaud. Sci. Nat..

[38] Schmieder R., Edwards R. (2011). Quality control and preprocessing of metagenomic datasets. Bioinformatics.

[39] Grabherr M.G., Haas B.J., Yassour M., Levin J.Z., Thompson D.A., Amit I., Adiconis X., Fan L., Raychowdhury R., Zeng Q. (2011). Full-length transcriptome assembly from RNA-Seq data without a reference genome. Nat. Biotechnol..

[40] Altschul S.F., Gish W., Miller W., Myers E.W., Lipman D.J. (1990). Basic local alignment search tool. J. Mol. Biol..

[41] Petersen T.N., Brunak S., von Heijne G., Nielsen H. (2011). SignalP 4.0: Discriminating signal peptides from transmembrane regions. Nat. Methods.

[42] Pedregosa F., Varoquaux G., Gramfort A., Michel V., Thirion B., Grisel O., Blondel M., Prettenhofer P., Weiss R., Dubourg V. (2011). Scikit-learn: Machine Learning in Python. JMLR.

