# Supplementary material for: Discovery of Novel Conotoxin Candidates Using Machine Learning
Source: Toxins (Basel). 2018 Dec 1;10(12):503. doi: 10.3390/toxins10120503 (PMC6315676; doi:10.3390/toxins10120503)
Supplement: Supplementary file 1 [file toxins-10-00503-s001.zip › toxins-372340-supple-final/toxins-372340-supplementary figures-final.docx]

Supplementary Materials: Discovery of Novel Conotoxin Candidates Using Machine Learning

Qing Li, Maren Watkins, Samuel D. Robinson, Helena Safavi-Hemami, and Mark Yandell

**Figure S1.** Box plot shows that the sensitivity varied among different combinations (single method, overlap or union of methods) of methods used. The union of different methods can achieve higher sensitivity. Union of methods means that the conotoxin is predicted by one method or another, as described in the caption for Figure 1.


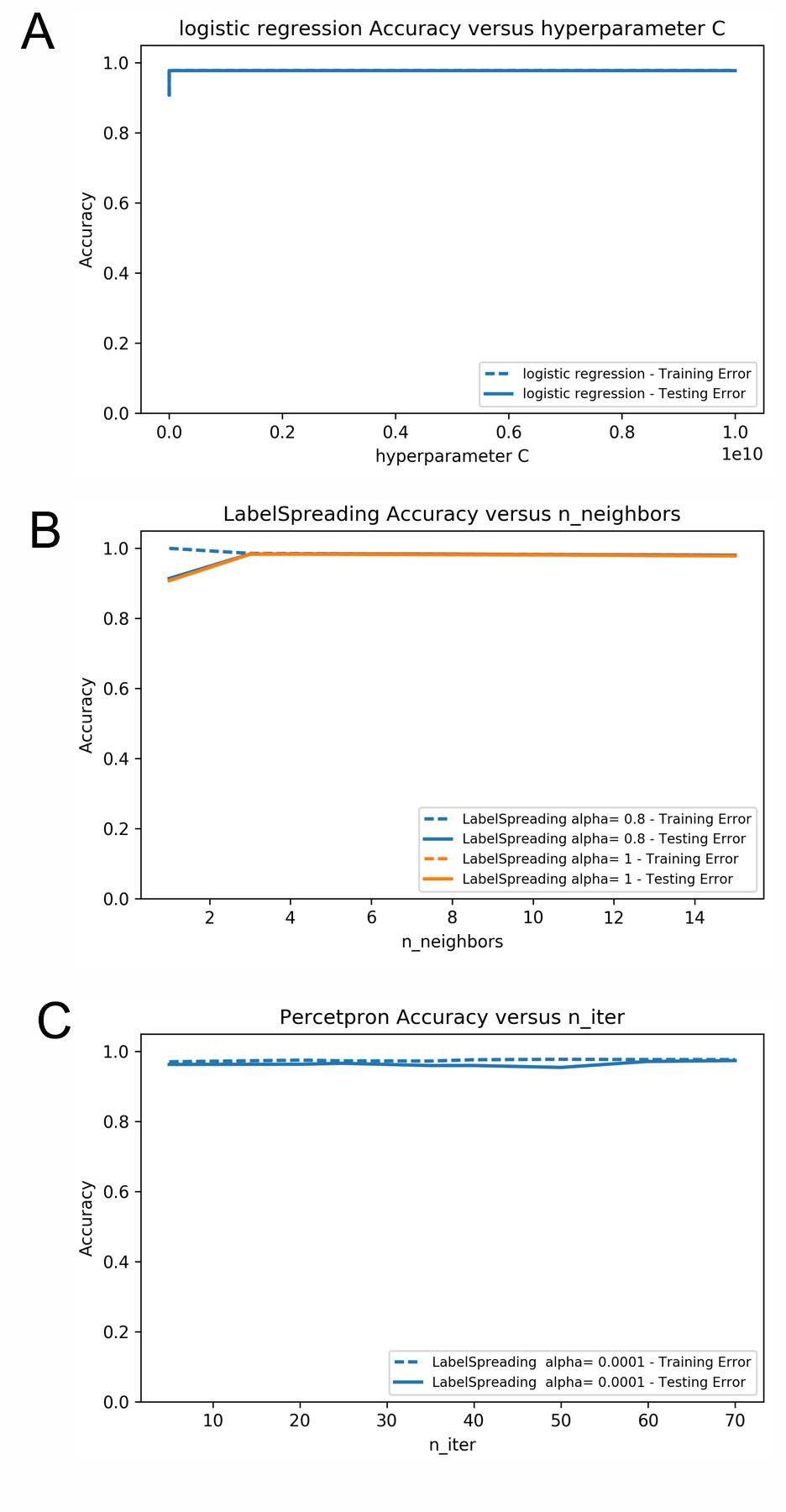


**Figure S2**. Plot of accuracy vs regularization parameter settings in each machine learning model. (**A**) Accuracy vs hyper-parameter C in logistic regression model. No overfitting/under fitting was observed. When choosing C = 10**10, the logit model achieved the best accuracy and sensitivity. (**B**) Accuracy vs hyper-parameter n_neighbors in LabelSpreading model. No overfitting/under fitting was observed. When choosing n_neighbors= 3 and alpha=1, the LabelSpreading model achieved the best accuracy and sensitivity. (**C**) Accuracy vs hyper-parameter n_iter in Perceptron model. Overfitting was observed between n_iter = 12 and n_iter = 25, between n_iter = 30 and n_iter = 50. When choosing n_iter = 5, the Perceptron model achieved the best accuracy and sensitivity.
